# Activated WNT signaling in postnatal SOX2-positive dental stem cells can drive odontoma formation

**DOI:** 10.1038/srep14479

**Published:** 2015-09-28

**Authors:** Guilherme M. Xavier, Amanda L. Patist, Chris Healy, Ankita Pagrut, Gabriela Carreno, Paul T. Sharpe, Juan Pedro Martinez-Barbera, Selvam Thavaraj, Martyn T. Cobourne, Cynthia L. Andoniadou

**Affiliations:** 1 Department of Orthodontics, King’s College London, UK; 2 Department of Craniofacial Development and Stem Cell Biology, King’s College London, UK; 3 Developmental Biology and Cancer Programme, Birth Defects Research Centre, Institute of Child Health, University College London, UK; 4 Department of Mucosal and Salivary Biology, King’s College London, UK

## Abstract

In common with most mammals, humans form only two dentitions during their lifetime. Occasionally, supernumerary teeth develop in addition to the normal complement. Odontoma represent a small group of malformations containing calcified dental tissues of both epithelial and mesenchymal origin, with varying levels of organization, including tooth-like structures. The specific cell type responsible for the induction of odontoma, which retains the capacity to re-initiate *de novo* tooth development in postnatal tissues, is not known. Here we demonstrate that aberrant activation of WNT signaling by expression of a non-degradable form of β-catenin specifically in SOX2-positive postnatal dental epithelial stem cells is sufficient to generate odontoma containing multiple tooth-like structures complete with all dental tissue layers. Genetic lineage-tracing confirms that odontoma form in a similar manner to normal teeth, derived from both the mutation-sustaining epithelial stem cells and adjacent mesenchymal tissues. Activation of the WNT pathway in embryonic SOX2-positive progenitors results in ectopic expression of secreted signals that promote odontogenesis throughout the oral cavity. Significantly, the inductive potential of epithelial dental stem cells is retained in postnatal tissues, and up-regulation of WNT signaling specifically in these cells is sufficient to promote generation and growth of ectopic malformations faithfully resembling human odontoma.

In humans the capacity to induce tooth formation *de novo* normally ceases after establishment of the secondary dentition^1^. In some circumstances supernumerary teeth can form, often manifesting during establishment of the secondary dentition and demonstrating wide morphological variation^2^. Amongst such malformations, odontoma represent a small group of hamartomatous malformations containing calcified dental tissues, which include both compound (containing numerous discrete structures faithfully resembling fully developed teeth) and complex (composed of a random assortment of poorly formed dental tissues) sub-types^3^,^4^.

Tooth development relies upon a series of reciprocal signaling interactions between oral epithelium (OE) and adjacent neural crest-derived mesenchyme^5^. WNT/β-catenin signaling is active at multiple stages of odontogenesis^6^ and pathway activation in embryonic OE, leads to the generation of ectopic teeth^7^. This has been demonstrated through expression of a constitutive-active form of *Ctnnb1*^7^,^8^ and through mosaic and constitutive deletion of the *Apc* inhibitor^9^. Conversely, pathway inhibition in OE or odontogenic mesenchyme can arrest tooth development^7^,^10^,^11^. In addition, WNT pathway activation throughout postnatal OE leads to ectopic tooth formation in both incisor and molar regions, depending upon age^9^,^12^. Collectively, these experiments have demonstrated the importance of appropriately regulated WNT signaling during murine odontogenesis; however, they have not identified the OE progenitor cell population through which, this pathway may act.

In many vertebrates, the transcription factor SOX2 marks multiple tissue-specific progenitor/stem cells (SCs), including in the embryonic dental lamina and postnatal incisor labial cervical loop^13^,^14^,^15^. *SOX2* expression has been detected in developing human dental epithelia and in third molar tooth germs from young adults^16^,^17^. Here, we have explored if SOX2-positive SCs are the cell type responsible for initiation of ectopic *de novo* tooth structures in postnatal OE. By genetically expressing constitutive-active *Ctnnb1* in *Sox2*-expressing SCs, we have modelled supernumerary tooth formation in the adult murine dentition; specifically, the formation of odontoma in the mandible and maxilla, and established that WNT pathway up-regulation specifically in dental SCs reinstates the inductive properties that occur during early tooth development.

## Results

### *De novo* odontogenesis following postnatal expression of constitutive-active Ctnnb1 in murine SOX2-positive SCs

**Figure 1:**
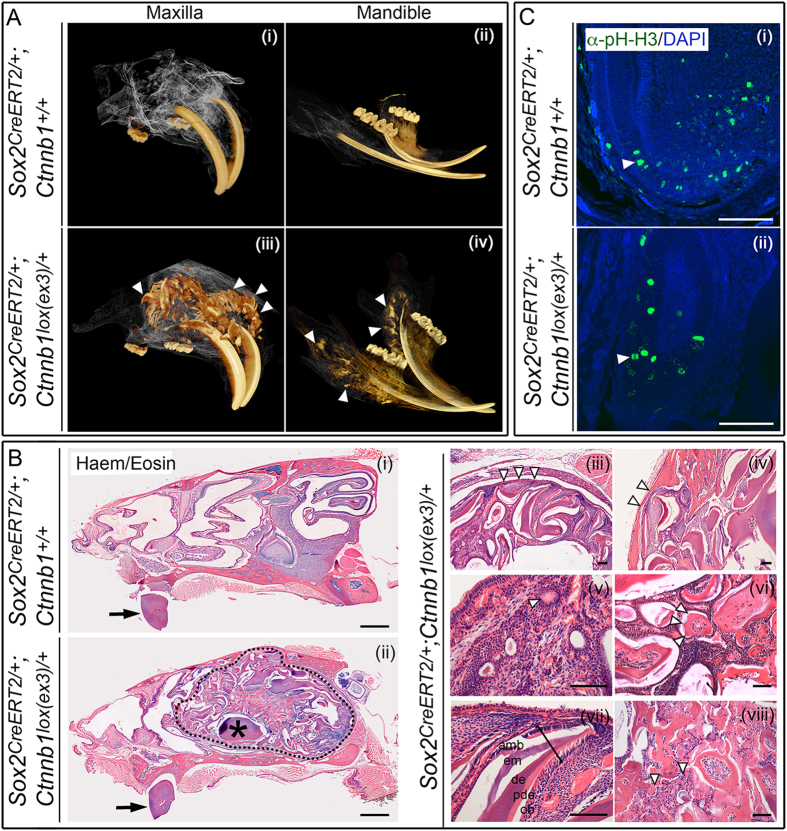
Postnatal expression of activated β-catenin in SOX2+ dental stem cells results in abnormal structures resembling odontoma. (**A**) Three-dimensional reconstruction of microCT scans of the maxilla and mandible of control *Sox2*^*CreERT2*/+^*; Ctnnb1*^+/+^ and mutant *Sox2*^*CreERT2*/+^*; Ctnnb1*^*lox(ex3)*/+^ animals at 5 months of age, 3 months after tamoxifen-induced CreERT2 activation. Mutant animals display multiple ectopic odontogenic structures of similar density to enamel and dentine, concentrated around the base of the incisors (arrowheads). (**B**) Haematoxylin and eosin-stained sagittal sections through a control decalcified head at 5 months of age (i), compared to a *Sox2*^*CreERT2*/+^*; Ctnnb1*^*lox(ex3)*/+^ mutant (ii–viii), 3 months following tamoxifen induction. Incisors are indicated by arrows and asterisk in (ii). Note the large malformation in the mutant outlined by dashed line in (ii). The asterisk denotes part of the normal incisor. Higher magnification images of malformations are shown in iii-viii. Arrowheads indicate the boundaries of malformation circumscription (iii), cortical thinning (iv), duct-like foci (v) and a tooth-like structure with multiple cusps (vi). Bracket in (vii) indicates normal orientation of epithelial and mesenchymal layers within a tooth-like structure (amb, ameloblasts; em, enamel matrix; de, dentine; pde, predentine; ob, odontoblasts). Irregular mineralization, osteodentine and dysplastic dentine, consistent with complex odontoma is shown in (viii), arrowheads. (**C**) Immunofluorescence staining with antibodies against phospho-histone-H3 to mark proliferating cells showing the normal proliferation pattern in the cervical loop of a normal incisor (i) and a similar pattern in an individual tooth-like structure from within the malformation (ii). Scale bars in B(i) = 1000 μm, B(ii–vii) and C = 100 μm.

We conditionally expressed an activated-mutant form of *Ctnnb1* in *Sox2*-expressing dental SCs in 5 week-old postnatal mice after the cessation of normal tooth induction using a tamoxifen-inducible *Sox2*^*CreERT2*/+^. Three-months following tamoxifen induction, microCT scanning revealed multiple calcified structures resembling ectopic teeth, in both the mutant maxilla and mandible (arrowheads in Fig. 1A). Not interfering with this phenotype, the formation of pituitary tumors in this model was previously described^18^. Histological examination confirmed a significant malformation surrounding the incisors in the mutant (dashed line in Fig. 1B-ii), which was well-circumscribed (arrowheads indicating malformation boundary in Fig. 1B-iii) and expansile, causing thinning of the maxillary cortex (arrowheads in B-iv). This malformation contained duct-like foci (B-v), with numerous single and multi-cusped tooth-like structures (B-vi) composed of dental tissue comprising normally orientated epithelial and mesenchymal-derived layers (pulp, odontoblasts, predentine, dentine, enamel matrix, ameloblasts, B-vii) and areas of irregular mineralization, resembling osteodentine and dysplastic dentine (B-viii). Taken together, these histological features resembled odontoma. The features that characterise these malformations in humans are a cell-rich zone of soft tissue with dentin and enamel, tubular or dysplastic dentine covered by enamel, tubular dentine enclosing circular or oval structures of mature enamel and irregular curvilinear clefts that contain enamel matrix-producing epithelium and connective tissue^19^. Phospho-histone-H3 staining revealed proliferating cells in both epithelial and mesenchymal compartments of ectopic teeth (Fig. 1C and^14^).

**Figure 2:**
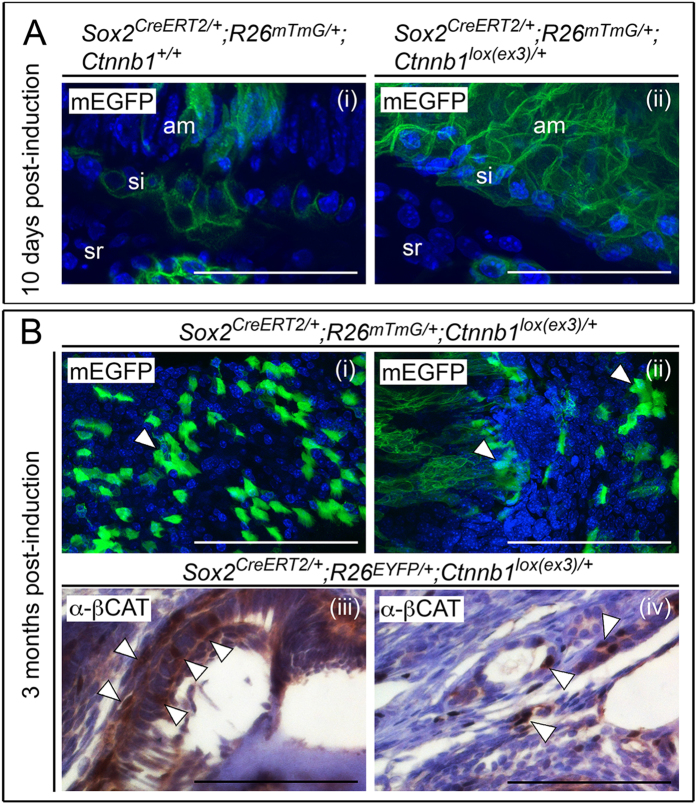
Genetic tracing of postnatal SOX2+ dental stem cells shows that structures resembling odontoma are heterogeneous in origin. (**A**) Lineage-tracing of SOX2+ cells at the base of the incisor by membrane-EYFP fluorescence in postnatal *Sox2*^*CreERT2*/+^*; R26*^*mTmG*/+^*; Ctnnb1*^+/+^ (i) and^*lox(ex3)*/+^ (ii) animals, 10 days following tamoxifen induction (sr, stellate reticulum; si, stratum intermedium; am, ameloblasts). Nuclei are counterstained with DAPI (blue). (**B**) Lineage tracing by fluorescence of membrane-EGFP positive cells in mature structures from malformations in *Sox2*^*CreERT2*/+^*; R26*^*mTmG*/+^*; Ctnnb1*^*lox(ex3)*/+^ mice, reveals a proportion of EGFP positive cells (green, arrowheads to positive clusters of cells, i-ii) as well as EGFP negative cells. Nuclei are counterstained with DAPI (blue). Staining with anti-β-catenin antibodies (α-βCAT) showing individual cells with nucleo-cytoplasmic accumulation of β-catenin (arrowheads, iii-iv), consistent with cells sustaining the β-catenin mutation. Sections are counterstained with haematoxylin. Scale bars = 50 μm in A, 100 μm in (**B**).

To characterize the site of odontoma initiation, we performed lineage-tracing of targeted cells following membrane-EYFP from the ROSA26 locus in *Sox2*^*CreERT2*/+^*; R26*^*mTmG*/+^*; Ctnnb1*^*lox(ex3)*/+^ animals. Ten days after tamoxifen-induced Cre activation, *Sox2*-descendants were identified in epithelia of the base of the incisor in control *Sox2*^*CreERT2*/+^*; R26*^*mTmG*/+^*; Ctnnb1*^+/+^ animals, as revealed by enhanced green fluorescent protein (EGFP) (Fig. 2A-i). Abundant GFP-positive cells were observed following mutant *Ctnnb1* expression in *Sox2*^*CreERT2*/+^*; R26*^*mTmG*/+^*; Ctnnb1*^*lox(ex3)*/+^ animals (Fig. 2A-ii). Analysis of mature malformations three months following Cre activation revealed that only a proportion of cells within the mass were GFP-positive (Fig. 2B-i-ii). Immunohistochemistry for β-catenin confirmed individual cells with strong nuclear expression (Fig. 2B-iii-iv), consistent with cells sustaining the β-catenin mutation.

### Constitutive WNT activation in Sox2-positive epithelium initiates expression of inductive developmental signals

**Figure 3:**
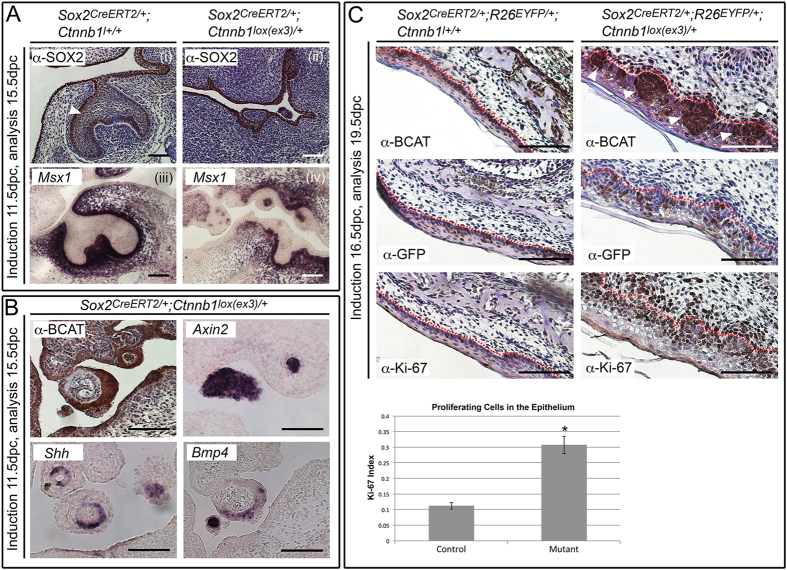
Embryonic induction of constitutive active β-catenin in SOX2+ embryonic progenitors reveals a non-cell autonomous mechanism of proliferation. (**A**) Tamoxifen induction at 11.5 dpc and analysis at 15.5 dpc reveals formation of multiple tooth buds in *Sox2*^*CreERT2*/+^*; Ctnnb1*^*lox(ex3)*/+^ embryos. Immunohistochemistry with SOX2 antibodies marks the epithelium in *Sox2*^*CreERT2*/+^*; Ctnnb1*^+/+^ control (i) and *Sox2*^*CreERT2*/+^*; Ctnnb1*^*lox(ex3)*/+^ mutant (ii). Note the uniform SOX2 distribution in the mutant, not exhibiting the stronger lingual expression observed in the control (arrowhead). Sections counterstained with haematoxylin. *In situ* hybridisation (ISH) with riboprobes against the BMP target *Msx1*, marks condensing odontogenic mesenchyme in control (iii), and mutant (iv) embryos, confirming *Msx1* expression surrounding the abnormal buds in the mutant. (**B**) *Sox2*^*CreERT2*/+^*; Ctnnb1*^*lox(ex3)*/+^ embryos show strong nucleo-cytoplasmic accumulation of β-catenin in foci, and aberrant expression of *Shh* and *Bmp4* as seen by ISH. Ectopic activation of the WNT pathway is confirmed through expression of *Axin2*. (**C**) Tamoxifen induction at 16.5 dpc and analysis at 19.5 dpc results in discrete foci accumulating β-catenin in the OE at the level of the second molar, in *Sox2*^*CreERT2*/+^*; R26*^*EYFP*/+^*; Ctnnb1*^*lox(ex3)*/+^ mice (arrowheads). Immunohistochemistry with anti-GFP antibodies (α-GFP) tracing the fate of CreERT2-expressing cells, revealing GFP positive cells within β-catenin accumulating regions (consecutive sections). Immunohistochemistry with antibodies against Ki-67 marks proliferating cells. Dotted red lines mark epithelial borders. Graph of epithelial mitotic index quantification in control (*Sox2*^*CreERT2*/+^*; R26*^*EYFP*/+^*; Ctnnb1*^+/+^) and mutant (*Sox2*^*CreERT2*/+^*; R26*^*EYFP*/+^*; Ctnnb1*^*lox(ex3)*/+^) embryos at 19.5 dpc. Error bars represent the standard deviation. Ki-67 increase is significant in mutants (unpaired *t*-test, *P *< 0.0001). Scale bars = 100 μm.

To determine the mechanism underlying novel tooth stimulation, we expressed activated-mutant *Ctnnb1* in embryonic OE at the dental placode stage, 11.5 dpc. This was sufficient to induce supernumerary structures resembling tooth buds at 15.5 dpc, interfering with normal tooth generation (Fig. 3A) and consistent with previous findings^7^,^8^,^9^. These structures were lined by SOX2-positive epithelial cells and surrounded by the expression of *Msx1* (Fig. 3A), a marker of condensing odontogenic mesenchyme^20^, previously shown to be dispensable during ectopic tooth induction^9^. We therefore established that the *Sox2*-expressing subpopulation of progenitor cells within the OE is responsible for this induction, in line with previous work determining that epithelial cells targeted to up-regulate the WNT pathway are capable of inducing surrounding mesenchymal and epithelial populations^9^. Immunohistochemistry for β-catenin identified foci of nucleo-cytoplasmic accumulation, resulting in discrete areas of active WNT signaling revealed by *Axin2* expression (Fig. 3B). These also expressed *Shh* and *Bmp4*, known signaling molecules that localize to the enamel knot of wild-type teeth (Fig. 3B)^21^ and are expressed in murine supernumerary teeth induced by activated WNT signaling throughout OE^7^,^8^,^9^.

We next expressed activated-mutant β-catenin at 16.5 dpc, the start of first molar cytodifferentiation. Established teeth remained unaffected but discrete foci of β-catenin accumulation formed in the OE by 19.5 dpc (Fig. 3C). Lineage-tracing of targeted cells in *Sox2*^*CreERT2*/+^*; R26*^*EYFP*/+^*; Ctnnb1*^*lox(ex3)*/+^ embryos demonstrated that the β-catenin-accumulating population included GFP-positive and GFP-negative cells as revealed by immunostaining using GFP antibodies recognising EYFP. The mutant epithelium appeared expanded and Ki-67 staining confirmed an increase in proliferation, with the majority of Ki-67-positive cells found outside the mutation-sustaining GFP-positive domain (Fig. 3C). These results support the idea that targeted *Sox2*-expressing SCs can induce neighbouring cells, resulting in an over-proliferative epithelium and morphological changes in mesenchyme, consistent with early tooth formation. Taken together, our data confirm that *Sox2*-expressing SCs are a cell type responsible for induction of the odontogenic programme, when ectopically targeted to up-regulate WNT/β-catenin signaling.

## Discussion

Through the conditional expression of an activated form of β-catenin in SOX2-expressing cells, we aimed to upregulate WNT signaling in multiple stem/progenitor populations in murine postnatal tissues throughout the organism. The targeted expression of mutant β-catenin in a SOX2-expressing subset of dental epithelial SCs, led to the induction of multiple supernumerary teeth and specifically, structures that resembled human odontoma. It has previously been demonstrated that activated WNT/β-catenin within murine OE can generate supernumerary teeth^7^,^8^,^9^,^12^. We provide evidence that pathway activation specifically in SOX2-positive progenitor/stem cells within the OE is sufficient to generate this phenotype in mice, providing a potential mechanism for the formation of odontoma in human populations.

Interestingly, genetic lineage tracing revealed that mature odontoma-like structures only had a partial contribution of cells derived from those sustaining the mutation, suggesting a paracrine induction of surrounding tissues. This is similar to findings from other tissues employing non-cell autonomous tumor-formation^18^,^22^,^23^ and indeed, supernumerary tooth formation in the mouse embryo achieved through K14-driven WNT activation in the OE^9^. The OE is instrumental in inducing odontogenic mesenchyme during tooth development and we confirm that the tooth-like structures do not derive solely from targeted SOX2-positive OE cells, but also from the surrounding mesenchyme. The expression of mutant β-catenin in OE leads to ectopic expression of inductive developmental signals (such as *Shh* and *Bmp4*), which influence the surrounding tissues and support aberrant mesenchymal proliferation and condensation. Significantly, mesenchymal contributions at postnatal stages, were also clearly identifiable with the presence of ectopic pulp, odontoblasts, predentine and dentine^9^,^12^. Consistent with this, ectopic tooth-like structures in human odontoma also contain tissues of both epithelial and mesenchymal origin^24^; specifically, odontogenic epithelium with odontogenic ectomesenchyme and dental hard tissue formation^19^.

In summary, we describe that sustained elevation of WNT signaling in murine dental epithelial SCs is sufficient to initiate *de novo* tooth formation in both embryonic and adult tissues. These data have implications for experimental tooth regeneration strategies and suggest a mechanism for odontogenic induction in the human postnatal dentition. Our findings clarify that mutated SOX2-positive SCs can underlie the pathogenesis of odontoma, through a mechanism of induction similar to embryonic odontogenesis, as previously described^9^,^12^.

## Methods

### Animals

Procedures were carried out in accordance with the UK Animals (Scientific Procedures) Act 1986, subject to KCL local Ethical Review. All experimental protocols were approved by the KCL Ethical Review Process Committee and the UK Home Office. *Sox2*^*CreERT2*/+^, *Ctnnb1*^*lox(ex3)*/+^ and *R26*^*EYFP*/+^ and *R26*^*membrane-Tomato;membrane-Green*/+^ (hereby *R26*^*mTmG*/+^) strains have been described^18^,^25^,^26^,^27^ and were maintained on a mixed CD1/C57Bl/6J background. For CreERT2 activation, 5 week-old males received a single tamoxifen injection (0.15 mg/g) (n = 11 per genotype) and for embryos, pregnant dams received 1.5 mg, co-injected with 0.75 mg progesterone (minimum n = 5 per genotype).

### Histology

Adult heads were fixed for 48 hours in 4% PFA at 4**°**C. Samples were decalcified in 14% EDTA for four months, dehydrated and stored in 70% ethanol at 4 **°**C. Wax embedding, sectioning and haematoxylin/eosin staining were carried out as previously described^28^,^29^.

### MicroCT

Specimens were immobilized using cotton gauze and scanned using a GE Locus SP microCT scanner to produce 14 μm voxel size volumes, using X-ray settings of 80 kVp, 118 uA and a 0.02 mm aluminium filter to attenuate harder X-rays. Three-dimensional isosurfaces were generated and measured using the dvanced region of interest and isosurface tools of the Microview software package (GE). Images were rendered using Drishti^30^.

### Immunostaining and *In Situ* Hybridisation

Paraffin sections at 8 μm were processed for immunofluorescence/immunohistochemistry, or mRNA *in situ* hybridisation using digoxigenin-labelled riboprobes as previously described^18^. For detection of EGFP from *R26*^*mTmG*/+^ animals, whole heads were fixed for 48 h in 10% neutral buffered formalin, washed in PBS and vibratome-sectioned at 50 μm. Sections were stained with DAPI and imaged on the confocal microscope, acquiring 16 sections through a 8 μm z-stack and processed for maximum projection using ImageJ. Mitotic-index was expressed as number of Ki-67-positive nuclei from total DAPI-positive nuclei in the epithelium (2500 cells counted from 5 sections/genotype). Statistical significance was determined by unpaired *t*-test.

## Additional Information

**How to cite this article**: Xavier, G. M. *et al.* Activated WNT signaling in postnatal SOX2-positive dental stem cells can drive odontoma formation. *Sci. Rep.* **5**, 14479; doi: 10.1038/srep14479 (2015).
